# A Retrospective Review on Severe Malaria in Colombia, 2007–2020

**DOI:** 10.3390/pathogens11080893

**Published:** 2022-08-09

**Authors:** Jaime Carmona-Fonseca, Mario J. Olivera, María F. Yasnot-Acosta

**Affiliations:** 1 Grupo Salud y Comunidad-Cesar Uribe Piedrahíta, Facultad de Medicina, Universidad de Antioquia, Medellín 050010, Colombia; 2 Instituto Nacional de Salud, Bogotá D.C. 111321, Colombia; 3 Grupo de Investigaciones Microbiológicas y Biomédicas de Córdoba (GIMBIC), Facultad Ciencias de la Salud, Universidad de Córdoba, Montería 230002, Colombia

**Keywords:** malaria, *Plasmodium*, *Plasmodium vivax*, incidence, Colombia

## Abstract

Background: Knowledge of severe malaria (SM) or complicated malaria is insufficient in all its components. The least known type is the one associated with *Plasmodium vivax*, compared to that caused by *P. falciparum*. The aim of this study was to provide a general overview of epidemiological information about the burden of SM, obtained from the National Public Health Surveillance System (SIVIGILA) for the period 2007–2020 in Colombia. Methods: A descriptive, retrospective, and cross-sectional study of secondary information was performed via SIVIGILA. Results: There were 9881 SM cases among 1,060,950 total malaria cases in Colombia in 2007–2020: 9.31 SM cases per 1000 malaria cases. During this period, there were 7145 SM cases due to the following species: *Plasmodium vivax*, 57.6%; *P. falciparum*, 38.6%; severe mixed malaria, 3.2%; and *P. malariae*, 0.6%. The most compromised organ systems are the hematological system (54.9%), the liver (9.1%), the kidneys (4.2%), the lungs (1.9%) and the brain (1.6%). Conclusions: There has been a reduction in malaria incidence in Colombia in the last 10–15 years, but there has also been a strong increase in SM incidence. We suggest emphasizing the prevention of the onset of severe malaria, with the early and accurate diagnosis of plasmodial infection.

## 1. Introduction

Malaria parasite propagation in the host affects several organs and modulates the functional outcomes of individual cells [1,2]. A few studies specifically focused on the description of SM in Colombia, but only one included all the cases in the country [3]. Before 2010, the criteria used to define SM in Colombia were the same as those established by the World Health Organization (WHO) for severe *falciparum* malaria, which were defined based on high-transmission areas. In 2010, the Colombian Ministry of Health (MoH) adapted these criteria to the Colombian transmission context, establishing more conservative parameters of complications to improve detection and ensure the more effective treatment of these cases. Some authors have questioned whether the severity criteria in the WHO guidelines are sufficiently sensitive to be used in clinical practice compared to national guidelines [4].

A 2020 Colombian Ministry of Health report shows that in 2018, there were 63,143 malaria cases, among which 61,880 were non-severe and 1263 were SM. Non-severe cases concerned the following species: vivax, 50%; falciparum, 48%; and both, 2%. SM cases presented the following species: *P. vivax*, 61.5%; *P. falciparum*, 34.6%; both, 3.9% [5]. This information indicates differences in the behavior of the parasitic species in non-severe malaria and in SM. 

The findings of an investigation with national coverage reported in 2016 [3] indicate that, between 2007 and 2013, a total of 547,542 cases of malaria were diagnosed and reported to SIVIGLIA; the cases corresponded to monoinfection by *P. vivax* (71.4%) and *P. falciparum* (27.4%), and mixed malaria by both species amounted to 1.2%. During the same period, 2553 (0.47%) cases of SM were reported. A total of 1274 (49.9%) of them were caused by *P. falciparum*, 1126 (44.1%) by *P. vivax*, and 153 cases (6.0%) were due to mixed malaria infections. In addition, four municipalities on the Pacific Coast with endemic malaria represented 21% of all complicated cases in the entire country [6].

**Figure 1:**
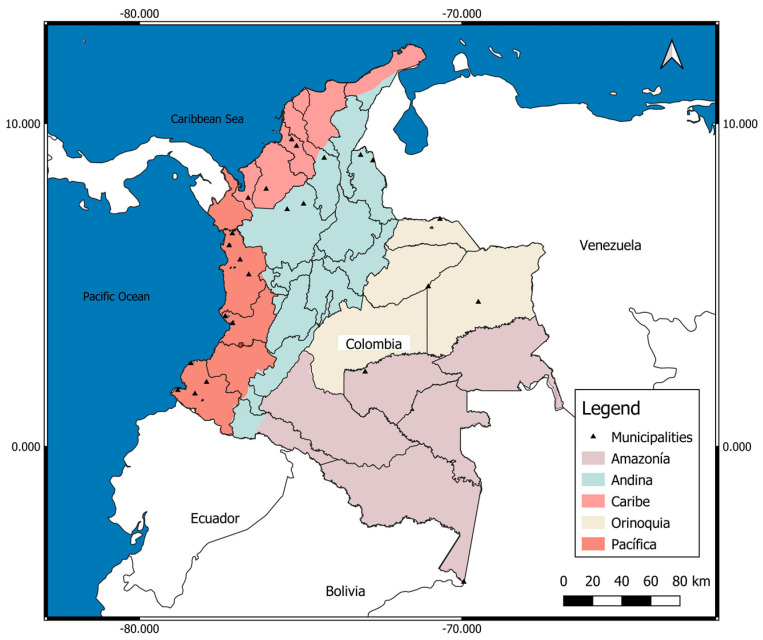
Political map of Colombia with its five natural regions. Pink: Atlantic/Caribbean Coast (Atlántica/Caribe) (132,288 km^2^; 10,301,982 inhabitants); departments (south to north): Córdoba, Sucre, Bolívar, Atlántico, Magdalena, Cesar, Guajira; Orange: Pacific Coast (Costa Pacífica) (excludes the part of the Andean mountains of the departments; 83,170 km^2^; 1,500,753 inhabitants); departments (north to south): Chocó, Valle Cauca, Cauca, Nariño; Beige: Orinoquia or Eastern Plains (254,335 km^2^; 1,840,922 inhabitants); departments (north to south): Arauca, Casanare, Vichada, Meta, Guainía, Guaviare; Violet: Amazonia (483,119 km^2^; 1,143,631 inhabitants); departments (north to south): Caquetá, Vaupés, Putumayo, Amazonas; Blue: Andean (Andina) (mountainous area, with a height greater than 1600 m above the sea; 282,540 km^2^ (3,068,593 inhabitants); departments (north to south): Norte de Santander, Santander, Antioquia, Boyacá, Cundinamarca, Caldas, Risaralda, Quindío, Tolima, Huila (includes the part of the Andean mountains of the departments). Source: Own elaboration on a map from the Agustín Codazzi Geographical Institute (IGAC) of Colombia. https://www.colombiaenmapas.gov.co/inicio/, accessed on 25 July 2022.

Colombia has five natural regions (Atlantic or Caribbean, Pacific, Andean, Orinoquia or Llanos Orientales, Amazonia) (Figure 1), each one very different from the others in terms of natural conditions, population (quantity, ethnic groups), socioeconomic development, and endemic and malaria transmission. For these reasons, the number of malaria cases, the risk of malaria, and the frequency of complications and death from malaria, among many other conditions, vary between natural regions and areas of a region [7,8,9].

Currently, few studies are published on the epidemiology of severe and non-severe malaria in Colombia. Therefore, it is necessary to direct studies towards a general overview of malaria, its natural and social context, information necessary to train health personnel responsible for patient care, researchers who carry out studies in endemic areas, and those responsible for formulating policies, epidemiology, and public health strategies, in order to reduce the impact of the disease in Colombia. The purpose of this study is to provide a general overview of epidemiological information about the burden of severe malaria (SM), obtained from the National Public Health Surveillance System (SIVIGILA) for the period 2007–2020 in Colombia.

## 2. Results

### 2.1. Malaria in Colombia in 2007–2020

**Figure 2:**
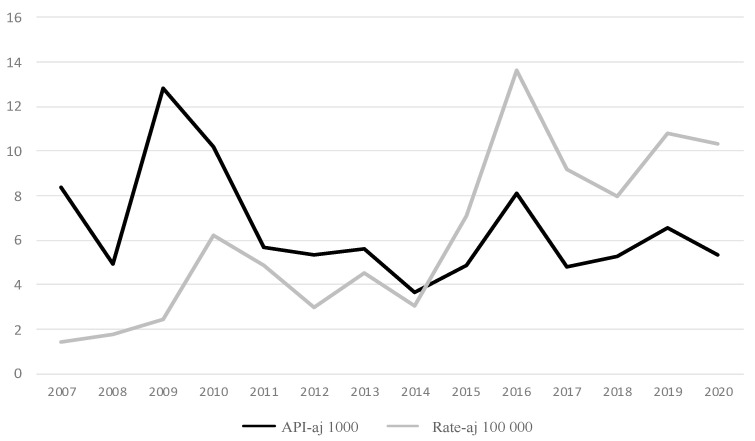
Total malaria and severe malaria cases in Colombia, 2007–2020: annual parasite index (API) of malaria and annual rate of severe malaria. API varies between 3.7 and 12.8 per 1000. Rate varies between 1.4 and 13.6 per 100,000. As of 2014, the rate of SM showed a sharp increase, while the API was relatively stable.

For the fourteen years of the period 2007–2020, 1,060,950 cases of malaria were registered in Colombia—that is, an average of 75,782 cases/year—with a clearly decreasing trend, both according to the cases (r = −0.465; slope: −6.37766 × 10^−5^) and the annual parasite index adjusted for the exposed population (APIaj per thousand exposed) (r = −0.437; slope: −0.729). This index has an average value of 6.540 for the studied period (Table 1, Figure 2).

### 2.2. Cases of Severe Malaria in Colombia 2007–2020

A total of 9881 cases were reported as SM malaria—that is, 0.931% (9881/1,060,950) or 9.31 SM cases per thousand malaria cases. Between 2007 and 2015 there were, on average, 429 SM cases/year, and between 2016 and 2020, this rose to 1203 cases/year, which indicates a 180% increase. Both the cases and the rate show an increasing trend (r = 0.835; slope = 0.008 for cases; r = 0.835; slope = 0.906 for rate) (Table 1).

Among the 9881 severe cases, 59.2% (5853/9881) occurred in men. In each sex, the average age is 25 years. It is found that 8186 cases were recorded with a known department of origin, for an annual average of 585 cases (8186 cases/14 years). The data by species are not available for the entire period of 2007–2014, but between 2015 and 2020, there were 7145 SM cases. The causal species were *Plasmodium vivax* (57.6%), *P. falciparum* (38.6%), both species cited (severe mixed malaria) (3.2%), and *P. malariae* (0.6%). *P vivax* predominated in each of these six years, with a minimum frequency of 48.9% in 2016 and a maximum of 64.2% in 2020, for an average of 57.6%. The average *vivax:falciparum* ratio was 1.5:1.0.

### 2.3. Risk of Malaria and SM in Colombia in 2007–2020

The adjusted API and adjusted rate are estimators of the risk of malaria and severe malaria in the exposed population. The national average of the adjusted API for malaria in 2007–2020 was 6.54 cases per thousand people exposed. The national average of the adjusted rate for SM in the same period was 6.17 per hundred thousand people exposed. In this period, 9.3 cases of SM occurred for every thousand cases of total malaria.

### 2.4. SM Risk in Colombia in 2015–2020 According to Year and Municipality

There were 270 municipalities that contributed to SM in 2015–2020; 24 municipalities reported 100 or more cases, which represented 4362 among the total of 7145 (61.0%); in other words, SM in was concentrated in 15 municipalities for the total of 1123 cases within the country, which represents 1.3% of them; the entire population of each is exposed to malaria. Data indicate 999.13 cases per 100,000 exposed in the Chalán municipality, in the Sucre department, located on the Atlantic Coast, while the value for Colombia as a whole is 59—that is, a ratio of 14.1:1.0. Compared to Colombia as a whole, the rates of Unión Panamericana, Orocué, Arauca, El Retorno, El Tarra, El Charco, Tiquisio, Litoral San Juan, and Bojayá are also very high (Table 2).

### 2.5. SM in 2015–2020 According to Residence Area, Ethnicity, Age, and Plasmodium Species

The place of residence was registered as the municipality center (center) in 43% of the 7145 cases accumulated in the period between 2015 and 2020; in another 43%, it was registered as “dispersed rural population”, and in the remaining 14%, it was described as a “rural population nucleus” (rural nucleus). The data refer to six ethnic groups (Black, 26%; Indigenous, 22%; Other, 52% (Rom, Raizal, and Palenquera), but “Other” is a denomination that does not allow further analysis, and the Rom, Raizal, and Palenquera ethnic groups only account for 0.4% (33 cases out of a total of 7145), so it is not possible to perform further analysis on these groups either. The place of residence for the ethnic groups is very different: 60% of the Indigenous group are dispersed rural populations, 59% of the Black group live in the municipality center, and “Other” groups are distributed as rural-dispersed populations and in the municipality center (45% and 40%, correspondingly). The age of the patients according to ethnicity varied between 16.9 in the Indigenous group, 25.0 in the Black group, and 29.4 in the “Other” group.

Six age groups (in years) are defined, and their frequencies of severe malaria cases are as follows: the age group of 0–14 years old accounts for 1934 cases (28.0% of the total 6897); the age group of 15–24 years old accounts for 1883 cases (27.3% of the total); the age group of 25–34 years old accounts for 1275 cases (18.5% of the total); the age group of 35–44 years old accounts for 818 cases (11.9% of the total); the age group of 45–60 years old accounts for 648 cases (9.4% of the total); and the age group of >60 years old accounts for 339 cases (4.9% of the total). The age differs statistically according to the parasitic species and ranges from 25.09 ± 17.7 years in *P. vivax* to 27.55 ± 17.5 years in the mixed infection group (*p*(KW) = 0.014).

*P. vivax* predominates in each population class: 51% in those located in municipality centers (i.e., urban malaria), 55% in rural areas, and 62% in dispersed rural areas. *P. falciparum* has the opposite distribution to that of *P. vivax*: 45% in municipality centers, 40% in rural nuclei, and 31% in dispersed rural areas. According to the ethnic group, parasites are distributed as follows: *P. vivax* in the “Other” group (62%) and in the Black group (25%), and *P. falciparum* in the Indigenous (44%) and in the “Other” (37%) groups. Among 409 pregnant women, *P. vivax* caused 46% of cases and *P. falciparum* caused 51%, compared to 54% and 42%, respectively, in 2556 non-pregnant women (*p*(X2) = 0.005).

### 2.6. Organs and Systems Affected by SM in Colombia in 2015–2020

The review of the organs and systems (O–S) affected by SM can be carried out in several ways, and one method is to evaluate the individual involvement of each O–S, without considering their combinations; thus, there are 6114 cases, representing 86% of the total 7145 cases; the other 1031 cases (14.4%) correspond to the involvement of two or more O–S. It can be said, then, that in Colombia, the most common situation is the alteration of an O–S and not of several simultaneously due to SM (ratio 5.9:1.0). The single most compromised O–S is the hematological system, which represents 3922 cases out of the total of 7145 (54.9%)—that is, more than half—followed by, very distantly, the liver with 651 cases, the kidneys with 304 cases, the lungs with 134 cases, and the brain with 113 cases. The group “others” (O–S other than the five mentioned above) contains 990 cases.

Table 3 shows the distribution of 7145 SM cases according to the O–S affected, either individually or as combinations of O–S. When considering the individual involvement of the hematic system (54.9%) plus its involvement combined with that of other O–S (11.5%), the participation of the hematic system reaches 66.4%. Therefore, it can be said that, in general, 2 out of 3 people with SM have the hematic system affected. The liver appears as the only compromised O–S in 9.5% and combined with others in 8.5%, for a total of 18%, which indicates that liver injury must be considered as often, as unique, or as associated with others. The kidney appears as the only compromised O–S in 4.3% of cases and associated with others in 4.8% (total: 9.1%). The lung is the only O–S injured in 1.9% of cases and it is associated with other O–S in 2.0% (total: 3.9%). The brain is the only O–S injured in 1.6% of cases and it is associated with other O–S in 1.3% (total: 2.9%).

The distribution of the O–S lesion by SM according to the *Plasmodium* species indicates that there is a significant association between O–S and *Plasmodium*, *p* (X2-Pearson = 0.001). *P. vivax* causes 58% of SM cases, *P. falciparum* causes 39% of SM cases, both species (mixed infection) cause 3%, and *P. malariae* causes 1%. In summary, Table 3 shows that in cases involving *P. vivax* and *P. falciparum,* the three most affected O–S are, in decreasing order, hematic, other, and hematic–hepatic; in *P. malariae* cases, these are hematic, hepatic, and hematic–hepatic, with very large differences between each one. The low involvement of the brain (between 1% and 3%) is striking, and the greatest involvement is generated by the mixed *vivax–falciparum* infection. Among the 4117 SM cases due to *P. vivax*, 1% are cerebral: among the 2758 SM cases due to *P. falciparum*, 2% are cerebral, and among 227 cases due to mixed SM, 3% are cerebral.

**Figure 3:**
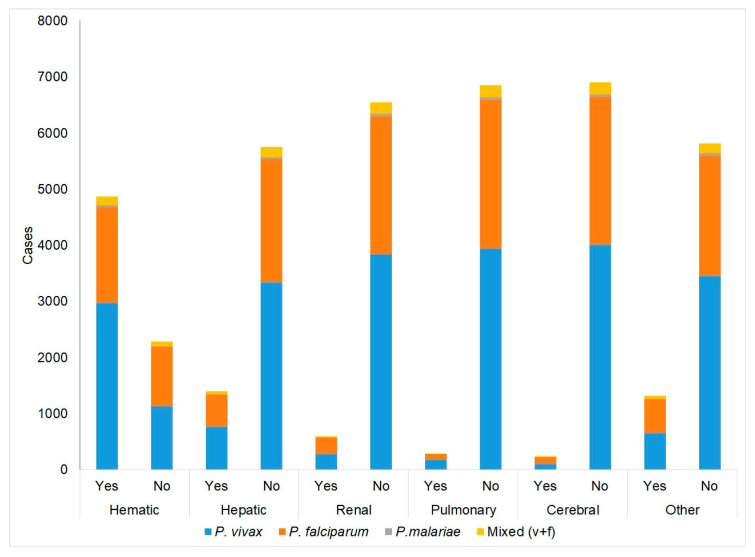
Cases of severe malaria: summary of all forms (alone or combined) according to *Plasmodium* species.

Grouping all SM cases into categories according to the parasite species, Figure 3 is obtained. Each SM form has a significant species association, except the hepatic *P. vivax* form, which predominates in hematic, hepatic, pulmonary, and other (50–61%) (Table 4). For the cerebral form, the predominance is given to *P. falciparum* (52% vs. 43%), and in the renal form, its weight is similar (47–49%). Considering only cerebral SM (as the only O–S affected or in combination with others), it is observed that there is a significant difference (*p* = 0.001) among the cerebral malaria proportions by species, and it is clear that the type caused by *P. falciparum* is the most frequent (52%), followed by *P. vivax* (43%), which together account for 95%. Mixed infection represents 227 cases (3.2%) and *P. malariae* accounts for 43 cases (0.6%).

The distribution of SM cases according to the sex of patients shows a significant association (*p* = 0.001) and indicates that 58% of SM cases occur in men; however, among them, there is an excess of SM cases of the hematic–renal types (67%), hepatic–renal types (69%), and hematic–hepatic renal types (70%), as well as little hematic–other involvement (42%). Renal complications (present, absent) and the sex of the patient showed a significant association (*p*(X2-Fisher = 0.000), but the other complications did not. Among the cases with renal injury, 64% (597/7145) occurred in men (*p*(X2-Fisher) = 0.001).

The median age according to the presence of complications (present, absent) exhibited a significant difference in the case of each complication, except the cerebral one (*p*(MW) = 0.000): older age was associated with the presence of complications for the cases of hepatic, renal, and pulmonary complications, and lower age in the case of hematic ones.

Hematic complications largely predominate in all age groups (between 54% and 60%), followed by “Other” complications (13% to 16%); then, liver complications (8% to 12%), renal complications (4% to 6%); pulmonary complications (1.2% to 2.7%); and cerebral complications (0.3% to 2.2%) change their order. The first one is the fifth most prevalent in the age groups of 1–14, 25–34, 35–44, and >60 years old, while cerebral complications are fifth in the groups of 15–24 and 45–60 years old (Table S1).

The location of residence for SM cases is a municipal center or dispersed rural area in 43% of cases, and a rural nucleus for 14%; hematic, hepatic, renal, and cerebral forms, but not pulmonary forms (alone or combined), have a significant association with the place of residence (Table 5).

## 3. Discussion

*Results interpretation*: while the general trend in malaria is decreasing for the period between 2007 and 2020, the trend for SM is increasing to an intense degree. Is this due to an improvement in the registration of cases, either through the application of diagnostic tests or in the information system, or is it because, in reality, the number of serious cases has increased? The answer is unclear, but it is possible that factors such as the poor state of laboratory infrastructure and the lack of professional personnel remain, in general, in the places where malaria is endemic within the country. In addition, the diagnostic criteria for SM are not modified to increase their sensitivity.

In 2010, the Colombian health authorities adapted the WHO guidelines on SM, particularly in terms of criteria thresholds, such as parasitemia, serum bilirubin levels, liver enzymes, and creatinine; but, the strong increase in cases and rate is taken into consideration, especially, as of 2015–2016. We suggest that the increase in Bajo Cauca, Chocó, the Pacific Coast, and the Amazon regions is due to different risk factors, such as illegal mining (gold, coltan), illegal forest logging, coca cultivation, and population displacement, which have seen a significant increase [10,11,12]. Likewise, in these malaria-endemic areas, patients are frequently treated by an individual within the community who has been trained in healthcare. However, they often have a low educational level and little supervision (Herrera, 2015), which may also represent a risk factor causing patients to develop severe malaria.

This fully agrees with the results of this analysis, which shows that, among the top six regions with the highest SM risk, there are four departments in the Amazon region, and the other two belong to the natural region of the Pacific Ocean Coast, where the aforementioned problems of illegal activities and population mobilization towards such sites are well known.

Speculation about urban malaria transmission in Colombia is not up to date, and some insist that it does not occur or has little importance [13], but the data in this report indicate otherwise. In fact, the place of residence for 43% of SM cases was registered as the municipality center, which is, without a doubt, exaggerated. These are, of course, the centers of small towns dispersed in the same areas referred to in the previous paragraph. These are municipalities such as Unión Panamericana and Litoral San Juan and Bojayá in Chocó; Orocué, Arauca, and El Retorno in the Orinoquia region; El Tarra in Norte de Santander; El Charco on the Pacific Nariño coast; Tiquisio in Southern Bolívar; and many others that constitute the list of some 200 towns that, having their populations settled in their centers and dispersed in the fields, have for decades been the main hubs of malaria cases in Colombia, as others have pointed out [7,8,14].

Given the above discussion, the distribution of SM cases according to ethnicity is consistent, in that cases are concentrated among the Black and Indigenous groups, accounting for 48%, since, in these places (Pacific Coast, Bajo Cauca in Antioquia, Amazon, Orinoquia regions), these ethnic groups constitute the majority of the population.

*P. vivax* appears as the parasitic species that predominates by far in SM in Colombia, although its incidence is lower than in general malaria (severe and non-severe cases), in which the ratio is 66% to 33% (2:1). This relationship perhaps suggests the higher pathogenic power of *P. falciparum*. The predominance of *P. vivax* in SM occurs both in the municipal centers and rural areas, and in the dispersed population in the “rural area” itself.

The proportion of SM by ethnic group shows an excessive contribution of two groups: “Black” populations (Black, Afro-Colombian, Raizal, and Palenquera) account for 26% of the cases and represent 9.34% of the total population (there are 4,671,160 people out of 50,010,706) and the Indigenous groups contribute 22% and account for 3.81% of the total cases (1,905,408). On the contrary, the “Other” ethnic groups represent 52% of SM cases and 86.85% of the Colombian population (43,434,298 out of 50,010,706).

In the SIVIGILA data review for Colombia between 2007 and 2013, other authors found, with the same SM case definition criteria that we apply, that “overall, hepatic and renal complications were the most common severe manifestations (63.6%)” [3]. It should be noted that in the list of complications used by the previous authors, the hematic–hematological category does not appear, despite the fact that SIVIGILA always includes the hematological category for SM in its reports in the Weekly Epidemiological Bulletin (BES, per its acronym in Spanish), and it always occupies first place. The aforementioned authors add that “whereas hepatic and pulmonary complications were more common in *P. vivax* infections, renal and cerebral complications were significantly more frequent in patients with *P. falciparum*” [3].

We found that the proportionally most affected O–S in cases caused by both *P. vivax* and *P. falciparum* are, in decreasing order, hematic, other, hepatic, renal, and pulmonary, and the only difference is in brain involvement, which is the sixth most commonly affected O–S in *P. vivax* (1%) and the fifth most common in *P. falciparum* (3%). These clinical findings are also corroborated by other studies in different countries. In a review by Baird and coworkers in 2013, they refer to studies that show serious pathological clinical states associated with a primary malaria diagnosis due to *P. vivax*. Studies describe *P. vivax*’s geographic distribution (India, Indonesia, Brazil, Venezuela, Colombia, Sudan, Pakistan, Afghanistan, Singapore, Uganda, Turkey, Honduras, Iran, Malaysia), showing a wide range of syndromes, with the exception of the low frequency of severe malarial anemia (SMA). Acute respiratory distress syndrome (ARDS), cerebral syndromes (seizures or coma), severe thrombocytopenia, hemorrhage, and shock syndromes are described in many of the patients [15].

A search for reports on SVM in Colombia, carried out in PubMed on 30 April 2022, used the expression “severe *vivax* malaria” and Colombia, identifying three articles with information on SVM, but only two that actually considered SVM. The expression “complicated *vivax* malaria” and Colombia retrieved one reference. The expression severe *vivax* malaria (without quotation marks) and Colombia retrieved 41 references, but only eight dealt with SVM.

These six articles are summarized in Table S2 [3,4,16,17,18,19,20,21]. In summary, the most frequent complications are as follows (gross percentages): hepatic with 24%, thrombocytopenia with 22%, renal with 19%, anemia with 12%, pulmonary with 8%, and cerebral with 7%. On the other hand, Foko and coworkers published in 2021 a meta-analysis of severe *vivax* malaria cases in India, describing an “atypical clinical spectrum” for SVM, finding, among others, thrombotic microangiopathy, myocarditis, and retinal hemorrhages; furthermore, the latter two were also reported in Brazil and South Korea [22]. In our study, based on data in Colombia, none of the “atypical clinical spectrum” was evidenced; however, special attention needs to be paid to these rare clinical findings, recently reported in all other areas where cases of severe *vivax* malaria occur.

In some of the studies that were found regarding severe malaria vivax in Colombia, it was observed that parasitemia is low (range between 100 and 2000 parasites/μL); unfortunately, in the data obtained from SIVIGILA, such information does not appear, but we believe that it is important to draw attention to this, given that there are cases of severe malaria due to *P. vivax* with low parasitemia [23]. It is known that despite the low parasite levels found in the peripheral blood, *P. vivax* causes high morbidity and may be associated with severe malaria and death [24]. In the different studies presented by Baird and coworkers in their review in 2013, there does not seem to be a clear correlation between the parasitemia burden and the disease severity, at least in the parasitemia scales that are typically applied to *P. falciparum*—for example, 200,000 p/ul, which carries a significant risk of poor patient outcomes [15].

On the other hand, the mechanism by which SM by *P. vivax* occurs is not clear; some researchers have postulated the following mechanisms: (i) sequestration in the bone marrow [25]; (ii) sequestration in the spleen, by the formation of reticulocyte-rich niches, where the parasites can cytoadhere and multiply while not circulating in the peripheral blood [26]; (iii) autoimmunity-mediating complications, such as atypical B cells producing antibodies against phosphatidylserine, which mediate pRBC destruction and, therefore, are involved in anemia [27], as well as exaggerated inflammation in the different organs due to immune complexes or hemozoin [28]. It is important and necessary to urgently carry out studies on these issues to clarify the severe malaria vivax pathophysiology.

The total SM cases caused by *P. malariae* amounted to 43 in 2015–2020, and 60% were registered in 2015, 21% in 2016, zero in 2017 and 2018, 2% in 2019, and 16% in 2020. This distribution seems to resemble that of epidemic outbreaks. The frequency of this parasite is very low even in malaria-endemic areas. In Colombia, there was news of an endemic focused on the Pacific Coast (Buenaventura municipality, Valle del Cauca, northern part of Cauca, and southern part of Chocó) [29], and, in recent years, this information was extended to the Amazonas department, where it has been found in the peripheral blood of 1392 symptomatic patients using PCR; the prevalence was around 39%, while the thick smear was negative in these patients [30,31]. An analysis of 387,993 samples derived from the entire country (except the Magdalena and Casanare departments) carried out in 2008 only found one *P. malariae* sample [32]. A meta-analysis published in 2020 that searched the world literature reported that “severe complications among patients with *Plasmodium malariae* infections are rare”, and this analysis demonstrates the global prevalence (3%) and mortality (0.17%) of severe *P. malariae* infection in humans. Severe anemia (3.32%), pulmonary complications (0.46%), and renal impairments (0.24%) were the most common severe complications found in patients with *P. malariae* infection. Data include mention of cerebral malaria (according to WHO 2014 criteria) for three *P. malariae* cases (0.03%) [33].

*Study results generalizability (external validity)*: External validity refers to the degree to which the results of a study can be generalized to other different populations. This validity, in the present study, could be affected by selection biases, and in order to control them, all the records that met the inclusion criteria were included; thus, this study is unlikely to be affected by this bias.

*Limitations*: We begin by acknowledging and pointing out the significant deficiencies in terms of epidemiological information in the data obtained and analyzed for this report. This information is completely lacking in clinical data. It is important and useful to point out that the SM case definition is based on criteria set forth in a Colombian National Institute of Health protocol [5], but it is not possible to diagnose SM in places without human and/or technical resources.

## 4. Materials and Methods

For the presentation of this report, the guidelines for the communication of observational studies, STROBE, were followed [34].

### 4.1. Study Location, Study Type, Sample Size

Data had national coverage (Colombia), by departments and municipalities, between 2007 and 2020 (Figure 1). A descriptive and retrospective study was conducted using data from the Colombian Public Health Surveillance System (SIVIGILA) for the 2007–2020 period. Severe malaria (SM) cases were captured and reported throughout the country, indicating each of the departments/states and municipalities where they were recorded.

### 4.2. Data Coverage (Sample Size)

Data represent all the records (*n* = 1,060,950) found in the National Public Health Surveillance System (SIVIGILA) [6]. The data are public, but with nonpublic access.

### 4.3. Variables Studied

All the variables described in this study were contained in two SIVIGILA databases. One database contained data between 2007 and 2019 and another between 2015 and 2020. The first database (2007–2019) contained the following variables: department, municipality, area of residence (urban, rural (hamlet, scattered)), gender (male, female), age, pregnancy status, and number of cases of severe malaria. Only age was a quantitative variable. The second database (2015–2020) contained the above variables plus others, such as *Plasmodium* species, pregnancy trimester, and affected organs or systems.

### 4.4. Study Participants

The malaria cases in Colombia were diagnosed by thick blood smear (TBS) and thin blood smear, which were analyzed by light microscopy. Some diagnoses in rural areas were carried out by rapid test (RDT); however, all diagnoses were confirmed by thick smear.

The tests were carried out at the health institutions or points-of-care (POC) (private or public system) by a “primary agent” trained and certified by the National Institute of Health (INS-Colombia), usually from the community, who is responsible for malaria diagnosis, notification, and treatment of uncomplicated malaria cases. Basic information on sex, age, place of residence, gestational status, parasitic species, and parasite count is collected at the POC. If the TBS result is positive, the patient receives antimalarial treatment; if he or she does not respond to treatment and begins to show signs of complications, he or she is referred to the local or closest hospital. If the same situation occurs in the latter, the patient is sent to a center with a higher complexity level. Uncomplicated malaria cases are reported on a weekly basis, while complicated cases and death cases are reported on a daily data basis [5,6]. This information was recorded in SIVIGILA and was analyzed and presented in our study.

### 4.5. Bias Control

All the cases of the databases obtained and analyzed were included, so there were no selection or information biases. Confounding bias was also absent because no causal relationship was analyzed.

### 4.6. Definition of Severe Malaria Cases

Severe malaria case definition was established by the Colombian MoH guidelines, as shown in Table 6 [5,6]. Complications are reported as discriminated in the SIVIGILA malaria notification forms: hepatic, renal, pulmonary, and cerebral complications.

### 4.7. Statistical Analysis

Databases were refined in agreement with SIVIGILA recommendations: (a) verification of data integrity, which includes calculating the proportion of “empty data” and “no information data” for each study variable in the notification form; only variables with integrity higher than 85% were included in the study analyses; (b) verification of the coherence of the data between variables (for example, in women who reported pregnancy, we confirmed their age). Sociodemographic and clinical characteristics of the SM cases were described. Continuous variables were analyzed using the mean and standard deviation or the median and interquartile range, depending on the normality of the variable’s distribution. Non-metric variables were analyzed using frequencies and percentages. For the comparison of qualitative variables (sex, area of residence, etc.), the Chi-square test or Fisher’s exact test were used, as appropriate. The comparison of quantitative variables (age, etc.) was performed with the Student *t*-test. Data analysis was performed with SPSS 27.0 and Epiinfo 7.0. A *p* value of <0.05 was considered statistically significant.

## 5. Conclusions

There has been a reduction in malaria incidence in Colombia in the last 10–15 years, but there has also been a strong increase in SM incidence. Severe malaria caused by *P. vivax* is more frequent than *P. falciparum* in our country, with complications being principally hematic, hepatic, and respiratory.

We suggest emphasizing the prevention of SM appearance, which consists, in essence, of performing early and accurate diagnosis of uncomplicated plasmodial infection. To this end, every day and in all scenarios, it is necessary to promote health consultation as soon as the symptoms appear, advise against self-medicating, provide diagnostic and treatment sites with the essential elements (microscopes, dyes, trained and supervised personnel), as well as provide appropriate medications. To the above, we must add health education, not as a fleeting action but as a stable program that is applied at the home/family, school, college, workplace, etc. This does not require high-cost or state-of-the-art technology.

## Figures and Tables

**Table 1:** Total malaria and severe malaria in Colombia, 2007–2020.

	Malaria ^a^		Severe Malaria ^a^	
Year	Cases	API-aj 10^3^	Cases	Rate-aj 10^5^
2007	110,389	8377	188	1427
2008	61,701	4957	217	1743
2009	149,999	12,826	288	2463
2010	115,884	10,185	706	6205
2011	62,716	5675	541	4896
2012	58,422	5337	324	2960
2013	60,383	5627	485	4519
2014	39,762	3664	334	3078
2015	53,254	4852	782	7125
2016	90,296	8135	1515	13,649
2017	52,954	4775	1015	9152
2018	62,141	5251	940	7943
2019	78,513	6559	1295	10,819
2020	64,536	5343	1251	10,356
	1,060,950		9881	
Total malaria				
	r	Slope b		
Cases	−0.465	−6.37766 × 10^−5^		
API 10^3^	−0.437	−0.729		
Severe malaria				
Cases	0.835	0.008		
Rate 10^5^	0.823	0.906		

^a^ API and rate are calculated based on exposed population.

**Table 2:** Twenty-four Colombian municipalities that contributed 100 or more total cases of malaria in the period 2015–2020, ordered according to the rate, from highest to lowest ^a^.

Municipality	Department; Region	Cases 5 y	Cases/Year	Population 2020	Rate 10^5^-aj
Chalán	Sucre; Costa Atlántica	230	46	4604	999.13
Unión Panamericana	Chocó; Cuenca del Atrato	156	31	6982	446.86
Orocué	Casanare; Orinoquia	230	46	12,652	363.58
Arauca	Arauca, Orinoquia	1667	333	96,814	344.37
El Retorno	Guaviare; Amazonia	128	26	13,722	186.56
El Tarra	Norte Santander, Andina	186	37	21,926	169.66
El Charco	Nariño; Costa Pacífica	186	37	22,550	164.97
Tiquisio	Bolívar; Bajo Magdalena	156	31	19,034	163.92
Litoral San Juan	Chocó; Costa Pacífica	186	37	22,890	162.52
Bojayá	Chocó; Cuenca del Atrato	100	20	12,326	162.26
Sucre	Sucre; Costa Atlántica	198	40	30,814	128.51
Alto Baudó	Chocó; Costa Pacífica	128	26	28,293	90.48
Ituango	Antioquia; Zona Andina	111	22	27,789	79.89
Istmina	Chocó; Cuenca del Atrato	111	22	30,806	72.06
El Bagre	Antioquia; Bajo Cauca	186	37	53,846	69.09
Leticia	Amazonas; Amazonia	1667	333	505,334	65.98
Timbío	Cauca; Costa Pacífica	116	23	36,287	63.93
Barbacoas	Nariño; Costa Pacífica	152	30	56,526	53.78
Tibú	Norte Santander; Catatumbo	156	31	58,721	53.13
Cumaribo	Vichada; Amazonia	198	40	78,863	50.21
Chigorodó	Antioquia; Urabá	127	25	59,836	42.45
Tumaco	Nariño, Costa Pacífica	493	99	257,052	38.36
Tierralta	Córdoba; Cuenca alta Sinú	116	23	95,177	24.38
Buenaventura	Valle Cauca, Costa Pacífica	298	60	311,827	19.11

^a^ The cases correspond to the 5-year annual average and the rate was calculated by dividing this average by the 2020 population; this gives an idea of the risk of severe malaria in each municipality.

**Table 3:** Severe malaria: organs and system affected in 7145 cases; Colombia 2015–2020.

Organ or System	Code of O–S	Organ–System Affected	*n*	%	Ac-%	Note
**Single organ or system**	1	Hematologic	3922	54.9	54.9	1 organ or system: 6114 cases; 86%
	2	Liver	651	9.1	64	
	3	Renal	304	4.3	68.3	
	4	Pulmonary	134	1.9	70.1	
	5	Cerebral	113	1.6	**71.7**	
	6	Other (no 1 to 5)	990	13.9	85.6	
**Hematic**	7	Hematic–Hepatic	351	4.9	90.5	2 organs or systems: 12%
	8	Hematic–Renal	89	1.2	91.7	
	9	Hematic–Pulmonary	42	0.6	92.3	
	10	Hematic–Cerebral	24	0.3	92.7	
	11	Hematic–Other	180	2.5	95.2	
**Hepatic**	12	Hepatic–Renal	82	1.1	96.3	
	13	Hepatic–Pulmonary	11	0.2	96.5	
	14	Hepatic–Cerebral	8	0.1	96.6	
	15	Hepatic–Other	31	0.4	97	
**Renal**	16	Renal–Pulmonary	8	0.1	97.1	
	17	Renal–Cerebral	10	0.1	97.3	
	18	Renal–Other	12	0.2	97.4	
**Pulmonary**	19	Pneumo–Cerebral	11	0.2	97.6	
	20	Pneumo–Other	11	0.2	97.7	
**Cerebral**	21	Cerebral–Other	6	0.1	97.8	
**3 O–S**	22	Hematic–Hepatic–Renal	103	1.4	99.3	3 organs or systems: 2%
	23	Hematic–Renal–Pulmonary	11	0.2	99.4	
	24	Renal–Pneumo–Cerebral	2	0	99.5	
**4 O–S**	25	Hematic–Hepato–Reno–Pulmonary	10	0.1	99.6	---
	26	Hepato–Renal–Pneumo–Cerebral	5	0.1	99.7	
**5 O–S**	27	Hemato–Hepato–Renal–Pneumo– Cerebral	24	0.3	100	---
		**Total**	**7145**	**100**	---	---

**Table 4:** Cases of severe malaria: organ system affected according to *Plasmodium*; Colombia 2015–2020.

O–S		*Plasmodium*					
		*P. vivax*	*P. falciparum*	*P. malariae*	*Pv + Pf*	Total	*p*(X2–Pearson)
		(*n* = 4117)	(*n* = 2758)	(*n* = 43)	(*n* = 227)	(*n* = 7145)	
**Hematic**	Yes	2980	1707	28	147	4862	0.001
	No	1137	1051	15	80	2283	
**Hepatic**	Yes	773	563	12	46	1394	0.182
	No	3344	2195	31	181	5751	
**Renal**	Yes	282	294	3	18	597	0.001
	No	3835	2464	40	209	6548	
**Pulmonary**	Yes	178	101	5	11	295	0.040
	No	3939	2657	38	216	6850	
**Cerebral**	Yes	103	126	2	11	242	0.001
	No	4014	2632	41	216	6903	
**Other**	Yes	657	599	10	49	1315	0.001
	No	3449	2155	32	178	5814	

**Table 5:** Cases of severe malaria according to place of residence; Colombia, 2015–2020.

O–S	Presence of O–S	Place of Residence (n; %)				*p*
		MH (*n* = 3053; 43)	RN (*n* = 1032; 14)	RD (*n* = 3060; 43)	Total (*n* = 7145; 100)	(X2–Pearson)
Hematic	Yes	2079; 43	635; 13	2148; 44	4862; 100	0.001
	No	974	397	912	2283	
Hepatic	Yes	655; 47	197; 6	542; 47	1394; 100	0.001
	No	2398	835	2518	5751	
Renal	Yes	288; 48	93; 16	216; 36	597; 100	0.003
	No	2765	939	2844	6548	
Pulmonary	Yes	118; 40	52; 18	125; 42	295	0.258
	No	2935	980	2935	6850	
Cerebral	Yes	118; 49	41; 17	83; 34	242	0.024
	No	2935	991	2977	6903	

MH: municipal head; RN: rural nucleus; RD: rural dispersed.

**Table 6:** Severe malaria criteria in Colombia.

Criteria	WHO, Defined before 2010	MoH, Defined after 2010
Cerebral malaria	Impaired consciousness or coma (Blantyre score < 3 or Glasgow score < 9); unconsciousness with the possibility of waking up	Impaired consciousness or coma (Blantyre score < 3 or Glasgow score < 9); unconsciousness with the possibility of waking up
Renal dysfunction	Serum creatinine > 3.0 mg/dL and/or urine vol < 400 mL in 24 h (adults) or <12 mL/kg of body weight in 24 h (children)	Serum creatinine > 1.5 mg/dL
Hepatic dysfunction	Serum bilirubin > 3 mg/dL and altered liver function tests	Serum bilirubin > 1.5 mg/dL or aminotransferases > 40 U/L
Respiratory distress	Increased respiratory rate at admission, presence of abnormal lung sounds or pulmonary edema (X-rays)	Increased respiratory rate at admission, presence of abnormal lung sounds or pulmonary edema (X-rays)
Circulatory collapse or shock	SBP < 70 mm Hg in adults or <50 mm Hg in children (3–5 years)	SBP < 80 mm Hg in adults
Hyperemesis	>5 episodes in 24 h	Not applicable
Hyperpyrexia	Axillary temperature > 39.5 °C	Not applicable
Hypoglycemia	Blood glucose level < 40 mg/dL	Blood glucose level < 60 mg/dL
Severe anemia	Hemoglobin < 5 g/dL or hematocrit < 15%	Hemoglobin < 7 g/dL
DIC	Abnormal bleeding in the presence of laboratory evidence of DIC	Abnormal bleeding in the presence of laboratory evidence of DIC
Acidemia/acidosis and hyperlactemia	Acidemia/acidosis (clinical signs)	Plasmatic bicarbonate < 15 mmol/L or base excess > −10; acidemia pH < 7.35; lactate acid > 5 mmol/L
Hemoglobinuria	Macroscopic hemoglobinuria	Macroscopic hemoglobinuria and positive urine dipstick
Hyperparasitemia	>100,000 asexual parasites/μL of *P. falciparum* or in mixed infection with *P. vivax* and schizontemia	>50,000 asexual parasites/μL

## Supplementary Materials

The following supporting information can be downloaded at: https://www.mdpi.com/article/10.3390/pathogens11080893/s1, Table S1: Organ systems affected according to age range; Table S2: Summary of six Colombian studies on severe vivax malaria.
