# Supplementary material for: A Retrospective Review on Severe Malaria in Colombia, 2007–2020
Source: Pathogens. 2022 Aug 9;11(8):893. doi: 10.3390/pathogens11080893 (PMC9416211; doi:10.3390/pathogens11080893)
Supplement: Supplementary file 1 [file pathogens-11-00893-s001.zip › pathogens-1750024-supplementary.pdf]

Supplementary Materials

Table S1. Organ-System affected according age range.

Code OSC	AGE RANGE (years)						
	1-14	15-24	25-34	35-44	45-60	>60	Total
1	1046	1017	752	433	344	176	3768
2	155	190	105	93	63	30	636
3	79	76	57	29	34	19	294
4	47	26	21	22	9	4	129
5	38	33	12	18	11	1	113
6	267	271	164	114	97	52	965
7	102	92	46	46	24	24	334
8	21	30	19	10	6	2	88
9	17	9	5	5	3	2	41
10	7	9	3	2	0	1	22
11	67	37	37	14	13	7	175
12	21	26	10	9	10	3	79
13	5	4	0	0	1	1	11
14	1	3	2	0	1	1	8
15	9	8	4	1	6	1	29
16	3	1	1	0	1	1	7
17	2	3	3	1	0	1	10
18	1	5	1	1	3	1	12
19	2	5	1	0	1	1	10
20	5	1	3	1	1	0	11
21	2	0	1	2	0	1	6
22	29	23	19	10	12	7	100
23	2	1	1	2	4	0	10
24	1	0	1	0	0	0	2
25	0	3	1	1	2	2	9
26	0	3	2	0	0	0	5
27	5	7	4	4	2	1	23
**Total of cases**	**1934**	**1883**	**1275**	**818**	**648**	**339**	**6897**

p(X2)-Pearson)= 0.038.

Table S2. Summary of six Colombian studies on severe vivax malaria.

					Complication; (%) [b]						
**Author**	**Region**	**Years of carry out the study**	**D[a]**	**Number of participants in the study**	**C1**	**C2**	**C3**	**C4**	**C5**	**C6**	**C7**
Tobón et al 2017	Turbo, Necoclí, El Bagre, Tumaco, Guapi, Timbiquí	1997-2007	R	193	H; 26%	A; 10%	T; 8%	R; 5%	C; 2%	P; 2%	HG; 1%
Chaparro et al 2016	Colombia	2007-2013	R	1126	H; 37%	R; 26%	P; 15%	C;11 %			
O´Brien et al 2014	Tumaco, Cali, Buenaventura	2009-2013	R	16	T; 25%	A; 22%	HB; 19%				
Tobón et al 2019	Antioquia (hospitals)	2010-2014	R	10	H [c]	A	Ac	T			

Arévalo-Herrera et al 2015	Tierralta, Quibdó, Tumaco	2011-2013	P	42	H; 4%	R; 15%	HU 20	P; 6,5%	A; 4%	T; 2%
Tovar-Acero et al 2021	South Córdoba	2017-2019	P	156	T; 54%	HG; 48%	H, K;30%			

[a] All designs are descriptive, cross-sectional; four are retrospective (R) and two are prospective (P); [b] Type of severe complication: H hepatic, A anemia, T thrombocytopenia, R renal, C cerebral, P pulmonary, HG hyperglycemia, HB hyperbilirubinemia, Ac acidosis, HU hemoglobinuria; [c] The report does not present a percentage of each complication in SVM. In summary, the most frequent complications are (gross percentages): Hepatic (24%); Thrombocytopenia (22%); Renal (19%); Anemia (12%); Pulmonary (8%); Cerebral (7%).
